# Pre-participation Cardiovascular Screening of Elderly Wrestlers

**DOI:** 10.5812/asjsm.34876

**Published:** 2010-03

**Authors:** Ali Vasheghani Farahani, Hossein Asheri, Saeed Alipour, Alireza Amirbeigloo

**Affiliations:** 1 Sports Medicine Research Center, Tehran University of Medical Sciences, Tehran, IR Iran; 2 Department of Cardiology, Tehran University of Medical Sciences, Tehran, IR Iran; 3 Department of Cardiology, Shahid Beheshti University of Medical Sciences, Tehran, IR Iran

**Keywords:** Wrestling, Cardiovascular disease, Elderly wrestler, Screening, Pre-participation examination

## Abstract

**Purpose:**

Sudden death of a competitive athlete is a tragedy that is usually caused by a previously unsuspected cardiovascular disease. The aim of this study was to clarify the role of noninvasive testing in pre-participation cardiovascular evaluation of elderly wrestlers.

**Methods:**

We included 63 Iranian elderly wrestlers who participated in Tehran international elderly wrestlers’ preparation camping by census method. A questionnaire including past medical and family history as well as coronary risk factors was filled out and then a complete physical examination of the cardiovascular system was done by an internist for all wrestlers. Electrocardiogram (ECG), complete echocardiographic examination and then symptom limited exercise test were performed and reported by the cardiologists who did not know the other examinations results.

**Results:**

Exertional dyspnea and typical chest pain (FC=I or II) were present in 5% and 1.7% of the examinees, respectively. There were one or more risk factors in 64.5% of the cases. Cardiovascular examination revealed abnormal heart sounds in 27.1%. ECG showed ischemic changes in 13.6% and premature atrial contractions and premature ventricular contractions in 11.4%. Echocardiography showed mild left ventricular systolic dysfunction in 3.4%, regional wall motion abnormality in 8.5%, valvular disease in 32.3%, diastolic dysfunction in 45.7%, and left ventricular hypertrophy in 16.9% of the cases. Exercise test results were negative, equivocal, positive and highly positive in 70.4%, 15.8%, 5.2%, and 8.6% of cases, respectively.

**Conclusion:**

Beside physical examination, pre-participation screening of elderly wrestling athletes with ECG and exercise testing is feasible and recommended in the presence of coronary risk factors or cardiac symptoms. Echocardiography can also be recommended to detect other relevant abnormalities when there is a clue in the standard history, physical examination or ECG.

## INTRODUCTION

Sudden death of a competitive athlete, a tragedy usually caused by a previously unsuspected cardiovascular disease, has given rise to several programs and panels for pre-participation cardiovascular evaluation of athletes all over the world^[1–5]^. The purpose of such screening is to provide medical clearance for pre-participation in competitive sports through routine and systematic evaluations intended to identify clinically relevant and pre-existing cardiovascular abnormalities and thereby reducing the risks associated with organized sports. However, detection of a possible cardiovascular abnormality on a standard screening examination is only the first step and referral to a specialist for further diagnostic investigations will probably be required. When a definitive cardiovascular diagnosis is made, a consensus panel should recommend participation in or disqualification from competitive sports. Early detection of clinically significant cardiovascular diseases through pre-participation screening will in many instances permit therapeutic interventions that may prolong life ^[6]^.

A variety of cardiovascular abnormalities represent the most common causes of sudden death in competitive athletes. However, lesions differ considerably with regard to age^[2–5, 7–10]^. For example, in youthful athletes (younger than 35 years), hypertrophic cardiomyopathy is the predominant abnormality in about one third of cases^[3, 4, 5]^. The next most frequent cause in this age group is congenital coronary anomalies, particularly anomalous origin of the left main coronary artery from the right sinus of Valsalva^[11, 12]^. These deaths occur most commonly in team sports such as basketball and football, which have the highest levels of participation^[6]^.

Older athletes (35 years and older) represent a different athletic population because they focus on individual endeavors such as long-distance running and wrestling. A vast majority of deaths in middle-aged athletes is caused by atherosclerotic coronary artery disease^[7, 8, 9, 10]^.

Sudden cardiac death in athletes is an infrequent event occurring in only a small proportion of participants. Indeed, the lesions known to be responsible for sudden death in younger athletes occur infrequently in the general population. These lesions range from relatively common anomalies, such as hypertrophic cardiomyopathy (1:500) to very rare abnormalities such as coronary artery anomalies, arrhythmogenic right ventricular dysplasia, and long QT syndrome, for which reliable estimates of frequency are lacking. Therefore, it is reasonable to estimate that congenital malformations relevant to athletic screening probably account for a combined prevalence of approximately 0.2% in athletic populations ^[6]^.

Although the prevalence of athletic field deaths is not known with certainty, it appears to be in the range of 1:100,000 to 1:300,000 in high-school-age athletes and is disproportionately higher in males ^[3, 4]^. Among older athletes, available estimates suggest that the frequency of sudden cardiac death principally due to coronary artery disease may exceed that of younger athletes (1:15,000 in joggers and 1:50,000 in marathon runners) ^[8, 13]^.

In the last decade attention to elderly wrestlers’ competitions has increased and several international elderly (31 to 50 years old) wrestling competitions took place. Currently there are no universally accepted standards for the pre-participation cardiovascular screening of elderly athletes. The aim of this study was to classify the role of noninvasive testing in pre-participation cardiovascular evaluation of elderly wrestlers.

## METHODS AND SUBJECTS

In this cross-sectional study which was conducted in 2006, we recruited all Iranian wrestlers who participated in Tehran international elderly wrestlers’ preparation camping by census method. We included 63 male wrestlers in this screening program. Of this, four individuals were excluded because of known cardiovascular diseases.

A questionnaire including complete information on past medical and family history as well as coronary risk factors was filled out by the subjects. Then a thorough physical examination of cardiovascular system including vital signs, heart and lung sounds and peripheral vascular exam was conducted by an internist for all wrestlers.

A 12-lead electrocardiogram (ECG) was reported by a cardiologist who did not know the wrestlers and their history and physical examination results. A complete echocardiographic examination was done for all wrestlers by a cardiologist, and then a symptom limited exercise test was taken in each case and reported by another cardiologist.

This study was approved by the Research Committee of Sports Medicine Research Center and Ethics Committee of Tehran University of Medical Sciences, Tehran, Iran.

## RESULTS

The age range of the wrestlers was from 37 to 78 years with a mean (±SD) of 54.42 (± 8.64) years. They had a history of wrestling from 12 to 55 years with a mean (±SD) of 37.76 (± 9.45) years. Fifty five wrestlers (93.2%) did not have any cardiovascular symptoms. Only three cases (5.1%) had exertional dyspnea and one subject (1.7%) had typical chest pain (FC=I or II). Twenty one cases (35.5%) did not have any coronary risk factors, but there were one or more risk factors in others including family history of coronary heart disease (25.6%), smoking (22%), hyperlipidemia (16.9%), hypertension (11.8%) and diabetes (5%).

Following the physical examination, 41 wrestlers (69.5%) were found to be completely normal. Abnormal findings were detected in 30.5% including abnormal heart sounds (27.1%), jugular venous distention (3.4%), and abnormal peripheral pulses (1.7%).

The 12-lead ECG was completely normal in 66.5%. Abnormal findings were ischemic changes in 13.6% that included pathologic Q wave in 3.4% and T inversion in 10.2%. There were premature atrial contractions and premature ventricular contractions in 11.4%, abnormal axis in 3.4%, first-degree atrioventricular (AV) block in 3.4%, and abnormal rhythm in 1.7% of the cases.

Echocardiographic findings consisted of normal ejection fraction (EF>55%) in 96.6% and mild LV systolic dysfunction in 3.4%. Regional wall motion abnormality was seen in 8.5% and 91.5% had normal wall motion. Valvular study showed 11.9% mild mitral regurgitation, 5.1% mild aortic regurgitation, 8.5% mild tricuspid regurgitation, 3.4% mild pulmonary regurgitation, and 3.4% calcified aortic valve. Doppler study of left ventricle showed 45.7% diastolic dysfunction (impaired relaxation) and 54.3% normal pattern. Left ventricular hypertrophy was reported in 16.9%, of the cases.

Exercise tests performed were negative in 70.4%, equivocal in 15.8%, positive in 5.2% and highly positive in 8.6%.

## DISCUSSION

Pre-participation cardiovascular screening using history taking and physical examination alone (without noninvasive testing) is not sufficient to guarantee the absence of many critical cardiovascular abnormalities in older trained athletes. Indeed, hemodynamically significant congenital aortic valve stenosis is probably the most likely lesion to be reliably detected during routine screening because of its characteristically loud heart murmur. Detection of hypertrophic cardiomyopathy by standard screening is unreliable, because most patients have the nonobstructive form of this disease, characteristically expressed by only a soft heart murmur or none ^[14, 15, 16, 17]^. Furthermore, most athletes with hypertrophic cardiomyopathy do not experience syncope or have a family history of premature sudden death due to the disease ^[3, 18]^.

The standard history taking and physical examination generally conveys low specificity for the detection of many cardiovascular abnormalities. In older athletes, however, a personal history of coronary risk factors can be useful for identifying the individuals at risk.

The 12-lead ECG has been proposed as a more practical and cost-effective alternative to routine echocardiography for population-based screening ^[19, 20]^. Indeed, the ECG is abnormal in about 95% of the patients with hypertrophic cardiomyopathy ^[21]^, and is frequently abnormal in other potentially lethal lesions such as coronary anomalies ^[12]^, and will usually identify the important but uncommon long QT syndrome ^[22, 23]^. Recent data indicate that a certain proportion of genetically affected relatives in families with long QT syndrome may have little or no phenotypic expression on ECG^[22]^. However, these problems are not common in older athletes. In pre-participation screening, ECG is compared unfavorably with the echocardiogram because of its lack of imaging capability for recognition of structural cardiovascular malformations. ECG also has a relatively low specificity as a screening test in athletic populations because of the high frequency of electrocardiographic alterations that are associated with normal physiological adaptations of an athlete's heart to training^[24]^. On the other hand, normal ECG cannot rule out coronary artery disease in older athletes.

In screening large populations of older trained athletes, the routine use of exercise testing to detect coronary artery disease is limited by its low specificity and pretest probability^[25]^. American College of Sports Medicine (ACSM) has recommended routine use of exercise testing for all male athletes over 40, female athletes over 50 and athletes with coronary risk factors. In athletes over 30 years old, coronary artery disease is an important problem, but real possibility of myocardial infarction (MI) and sudden cardiac death during exercise is low; therefore, the routine use of exercise testing is not recommended except when there is a cardiac symptom such as chest discomfort or when there are coronary risk factors^[26]^. In a study on 102 unprofessional football players with an average age of 45.5 years old, exercise test was positive in 52%^[27]^.

Echocardiography can also be expected to detect other relevant abnormalities associated with sudden death, such as valvular heart disease, aortic root dilatation, and left ventricular dysfunction (with myocarditis and dilated cardiomyopathy) in addition to regional wall motion abnormality due to coronary artery disease in older athletes. However, even such diagnostic testing cannot by itself guarantee the identification of all important lesions, and some diseases that may not be detectable with any screening method ^[28]^.

The potential false-positive or false-negative results is another important limitation of screening with two-dimensional echocardiography. False-positive results may arise from the assignment of borderline values for left ventricular wall thicknesses (or particularly large values for cavity size) that require formulation of a differential diagnosis between the normal physiological adaptations of an athlete's heart ^[29, 30, 31]^ and pathological conditions such as hypertrophic cardiomyopathy or other cardiomyopathies ^[33]^. In fact, such clinical dilemmas (which cannot be definitively resolved in some athletes) generate heavy emotional, financial, and medical burdens for the athletes, their families and the team for performing additional testing.

Cost-efficiency issues are important in assessing the feasibility of screening large athletic populations ^[33]^. There have been relatively few published reports of cardiovascular screening efforts in large older athletic populations so far. Most of the studies on athletes have implemented noninvasive testing (i.e., conventional or limited echocardiogram or 12-lead ECG) in high school or collegiate athletes. We hope that our study helps to clarify the role of noninvasive testing (12-lead ECG, echocardiography and exercise testing) in elderly athletes.

## CONCLUSION

Pre-participation cardiovascular screening of elderly wrestlers can be started by a standard history taking, physical examination and ECG. Then, exercise testing is recommended when there is either a cardiac symptom such as chest discomfort or when there is a coronary risk factor. Echocardiography can also be recommended to detect other relevant abnormalities associated with valvular heart disease, aortic root dilatation, and left ventricular dysfunction (with myocarditis and dilated cardiomyopathy) as well as regional wall motion abnormalities due to CAD when there is a clue in the standard history, physical examination, or ECG.
